# Replacing red and processed meat with lean or fatty fish and all-cause and cause-specific mortality in Norwegian women. The Norwegian Women and Cancer Study (NOWAC): a prospective cohort study

**DOI:** 10.1017/S0007114523002040

**Published:** 2024-02-14

**Authors:** Torill M. Enget Jensen, Tonje Braaten, Bjarne Koster Jacobsen, Daniel Borch Ibsen, Guri Skeie

**Affiliations:** 1 Department of Community Medicine, Faculty of Health Sciences, UiT The Arctic University of Norway, NO-9037 Tromsø, Norway; 2 Centre for Sami Health Research, Department of Community Medicine, Faculty of Health Sciences, UiT The Arctic University of Norway, Tromsø, Norway; 3 Steno Diabetes Center Aarhus, Aarhus, Denmark; 4 Department of Public Health, Aarhus University, Aarhus, Denmark

**Keywords:** Red and processed meat, Lean fish, Fatty fish, Substitution analyses, Cause-specific mortality

## Abstract

Nordic Nutrition Recommendations recommend reducing red and processed meat and increasing fish consumption, but the impact of this replacement on mortality is understudied. This study investigated the replacement of red and processed meat with fish in relation to mortality. Of 83 304 women in the Norwegian Women and Cancer Study (NOWAC) study, 9420 died during a median of 21·0 years of follow-up. The hazard ratios (HR) for mortality were estimated using Cox proportional hazards regression with analyses stratified on red and processed meat intake due to non-linearity. Higher processed meat (> 30 g/d), red and processed meat (> 50 g/d), and fatty fish consumption were associated with higher mortality, while red meat and lean fish consumption were neutral or beneficial. Among women with higher processed meat intake (> 30 g/d), replacing 20 g/d with lean fish was associated with lower all-cause (HR 0·92, 95 % CI 0·89, 0·96), cancer (HR 0·92, 95 % CI 0·88, 0·97) and CVD mortality (HR 0·82, 95 % CI 0·74, 0·90), while replacing with fatty fish was associated with lower CVD mortality (HR 0·87, 95 % CI 0·77, 0·97), but not with all-cause or cancer mortality. Replacing processed meat with fish among women with lower processed meat intake (≤ 30 g/d) or replacing red meat with fish was not associated with mortality. Replacing processed meat with lean or fatty fish may lower the risk of premature deaths in Norwegian women, but only in women with high intake of processed meat. These findings suggest that interventions to reduce processed meat intake should target high consumers.

Red meat mainly refers to meat derived from pork, cattle, sheep and goat^(1,2)^. Processed meat primarily consists of red meat that has undergone modifications like curing, salting, or smoking and often contains minced fatty tissues. It includes items such as bacon, sausages, ham, salami, liver pate and similar products^(1)^. Red meat is an important source of energy and nutrients such as proteins, essential amino acids, vitamin B_6_, vitamin B_12_, Zn and Fe^(1)^. However, red meat, especially processed meat, is also a significant source of SFA and of substances formed during processing that can have adverse effects on health^(1,3)^.

There is strong evidence that processed meat consumption increases the risk of colorectal cancer, and probable evidence that red meat consumption also increases the risk^(4,5)^. Red meat, and particularly processed meat, is a probable risk factor for type 2 diabetes and CVD, which are leading causes of death in high-income countries^(6–9)^. The evidence indicates that the association with mortality is stronger and more consistent for processed meat compared with red meat^(9)^. The precise mechanisms underlying the adverse health effects linked to the consumption of red and processed meat are not yet fully established^(1,3)^. However, the presence of saturated fats and heme iron, in addition to Na and processed induced substances such as heterocyclic aromatic amines, and lipid peroxidation products, have been proposed to contribute to the increased mortality and disease from processed meat consumption compared with red meat consumption^(1,3)^.

Reducing the intake of red and processed meat, as recommended by dietary guidelines, must however be compensated by an increased intake of other energy-contributing foods to maintain a balanced energy intake^(1,10)^. Fish serves as viable alternative to red and processed meat, providing high-quality protein and essential nutrients such as vitamins A and B_12_, Fe, and Zn^(2)^. Additionally, fish has a low content of SFA and is a source of the long-chain *n*-3 fatty acids, EPA and DHA, I, Se, and vitamin D^(2)^.

Increasing fish intake while reducing red and processed meat consumption could have potential benefits for public health, but there are only a few studies that have specifically examined the implications of this replacement on mortality in specified substitution analyses^(11–14)^. While these studies found lower mortality by replacing red and/or processed meat with alternative sources of protein, including fish, they did not differentiate between replacement of red and processed meat with lean or fatty fish. Findings from the Norwegian Women and Cancer Study (NOWAC) study indicates that a higher consumption of lean fish could have potential benefits in relation to all-cause mortality, whereas lower intake of fatty fish showed a neutral association with all-cause mortality, and higher intake was linked to higher all-cause mortality^(15)^. Another NOWAC study found that lean fish consumption, but not fatty fish, was associated with lower risk of type 2 diabetes mellitus, suggesting that distinguishing between types of fish is important when examining associations with cause-specific mortality^(16)^.

When conducting analyses using specified food substitution models, there is an assumption that the relationship between exposure and outcome(s) is linear. While there is evidence supporting a linear relationship between red and processed meat consumption and mortality^(9)^, there are also indications of potential non-linear associations^(7,8,17,18)^.

Therefore, the main objective of this study was to investigate how replacing red and processed meat with lean or fatty fish is associated with all-cause mortality, and mortality related to cancer and CVD (ischemic heart disease (IHD) and stroke), within a cohort of Norwegian women. In support of the main objective, the study aims to consider potential non-linear associations between red and processed meat and fish consumption and cause-specific mortality outcomes, as well as the associations between red and processed meat and fish consumption and mortality outcomes without the substitution.

## Methods

### Study population

We used data from the NOWAC study, including women who have answered a questionnaire about different lifestyle factors, in particular food frequency questions. Data were collected in the period between 1996 and 1998 or 2003 and 2005, from women aged between 41 and 70 years at inclusion. Women were randomly selected from the National Registry of Statistics Norway^(19)^. The study sample has been found to be representative as no major source of selection bias was revealed in a study assessing the external validity of the NOWAC cohort^(20)^. The study found minor differences between responders and the total sample regarding education and parity, but no significant differences in relation to cancer incidence rates.

**Fig. 1.**
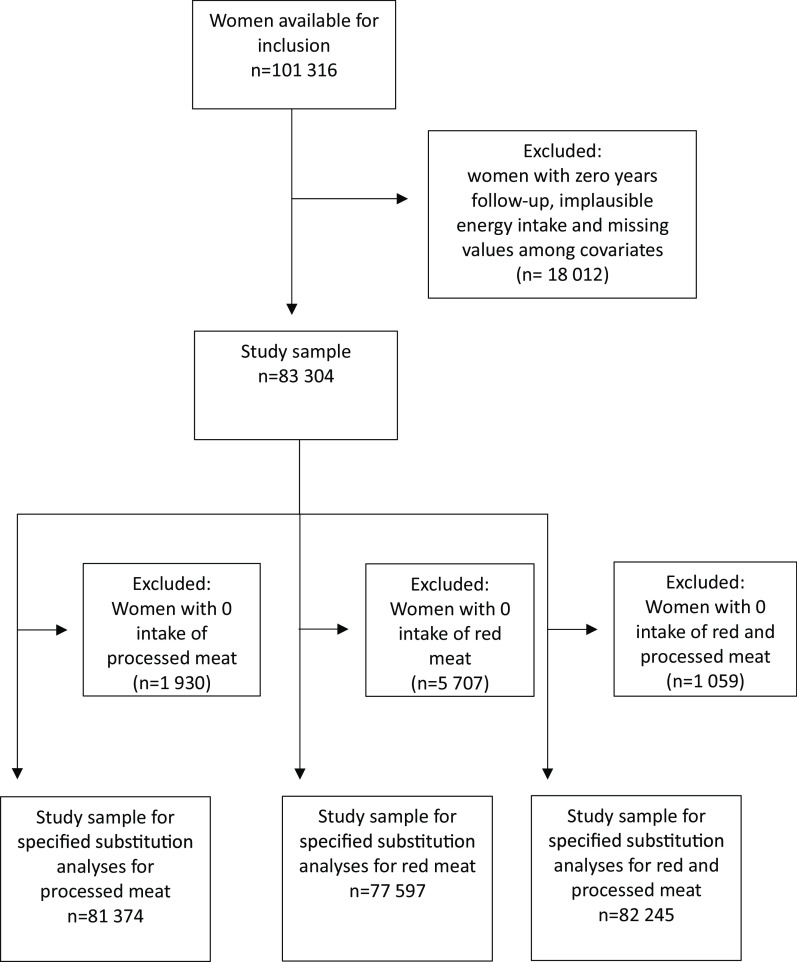
Flow chart with overview of participants included in the analytic samples.

A total of 101 316 women were available for inclusion in this study. Women with zero person-years of follow-up (*n* 20), implausible energy intake (< 2500 kJ/d (*n* 1053) or > 15 000 kJ/d (*n* 140)), and missing values for the covariates of physical activity (*n* 8539), education (*n* 4684), smoking (*n* 1306) and BMI (kg/m^2^) (BMI) (*n* 2270) were excluded from the analytical sample. A total of 83 304 women were included in the analyses for lean and fatty fish consumption and mortality, while non-consumers of processed meat (*n* 1930), of red meat (*n* 5707) and of red and processed meat (*n* 1059) were excluded in the analysis of red and processed meat and mortality outcomes and in the substitution analyses, respectively; see Fig. 1 for clarification.

The NOWAC cohort received approval for the collection and storage of the questionnaire information. All data were stored and handled according to permission provided by the Norwegian Data Protection Authority (Ref.nr. 07–00030). Participants provided written informed consent, and ethical approval for the NOWAC cohort was obtained from the Regional Committee for Medical and Health Research Ethics (REK) (Ref.nr. 200300119–5).

### Exposure

Dietary data were collected using validated semi-quantitative FFQ which were developed to measure usual food intake over the past year^(21–23)^. The respondents were asked to report the average food consumption in four to seven frequency categories ranging from never/seldom to six or more per week. The FFQ have been slightly improved and adapted as new hypotheses have been generated, new products have been introduced, and other products have been removed from the market during the data collection period of almost 10 years. In total seven, grouped into five for stratification, slightly different versions of the FFQ part of the lifestyle questionnaires have been used to collect dietary data in this cohort. The items included in the FFQ varied from approximately seventy-three to ninety frequency questions, but most of the questions used to estimate the exposures in this study have remained consistent over time^(24)^. In addition to the frequency questions, there were separate portion size questions for most fish, meat and fish and meat products consumed as main dishes. For sandwich spreads, participants reported how many slices of bread they consumed with the various spreads, and this was multiplied with standard portions^(25)^. To account for small variations between different versions of the FFQ, those which were completed closest together in time were grouped together in subcohorts (*n* 5), and subcohorts were used as a stratification variable as per NOWAC analytical strategy^(24)^.

In this study, red meat included beef, chops and roast, and processed meat included sausages, meatballs/burgers, and sandwich meat made from red meat (not including processed poultry) but excluded red and processed meat as part of combined dishes, such as pizza and stew. Lean fish included cod, saithe, haddock, plaice, catfish, flounder, redfish, fish cakes, fried fish and tuna in oil/water but excluded lean fish as part of other combined dishes. Fatty fish included salmon, trout, herring, mackerel, mackerel spread, sardine in oil, pickled herring, smoked and cured salmon but excluded fatty fish as part of other combined dishes. Subtypes of fish or fish products, which could not be defined as lean or fatty fish such as ‘other fish’, shellfish, liver, caviar and roe were not included in the lean or fatty fish exposures but were rather controlled for in the analyses. Red and processed meat and lean and fatty fish were expressed as continuous exposures with 20 g/d increments in the analyses, and substitutions of red and processed meat with lean or fatty fish were expressed in servings of 20 g/d.

The daily intake of food and energy was calculated for each participant by converting consumption frequency and portion size to g/d, based on information about standardsed portion sizes and weights obtained from the Norwegian Weight and Measurement Table^(25)^, and information about nutrient content in foods obtained from the Norwegian Food Composition Database^(26)^. The calculations were done using a statistical syntax in SAS (SAS Institute Inc., Cary, NC, USA), developed at the Department of Community Medicine, UiT The Arctic University of Norway, for the NOWAC cohort.

### Outcomes

The outcomes of interest were all-cause mortality and death due to cancer and the major subtypes of CVD of which atherosclerosis is a common risk factor, that is, IHD and stroke. Mortality outcomes were defined according to the International Classification of Diseases, 10th Revision codes: cancer including malignant neoplasms at all sites (C00-C97), CVD including IHD (I20-I25) and stroke (I60-I69). To obtain information on death, the NOWAC study participants were linked to the Norwegian Cause of Death Registry using the unique personal identity number. Participants were followed up until the date of emigration or death or 31 December 2019, whichever came first.

### Covariates

Included covariates were chosen *a priori* based on literature and directed acyclic graphs (online Supplementary Fig. 1).

Information on age (years) was based on information from the National Population Registry in Norway, whereas all the other covariate information was obtained from the lifestyle questionnaires (which included the FFQ). The variable for physical activity was based on self-reported physical activity levels on a scale from low (1) to high (10), including physical activity at home, work, exercise and walking^(27)^. The smoking variable was computed by combining information about smoking status (never, former and current), age at smoking initiation and the number of pack-years (number of cigarettes smoked per d, divided by 20, multiplied by the number of years smoked). Information on education was based on self-reported number of years of schooling. Total energy intake, excluding energy from alcohol (kJ/d), alcohol intake (g/d) and other foods (g/d), were obtained from the FFQ.

BMI was calculated as weight divided by the square of height based on validated self-reported weight (kg) and height (m)^(28)^. Information about prevalent diabetes (yes/no) was self-reported and obtained from lifestyle questionnaires^(16)^.

### Statistical analyses

Descriptive statistics were used to calculate baseline characteristics for the total cohort and for low and high consumers of processed meat and low and high consumers of red meat, using proportions for categorical variables and medians and 10th and 90th percentiles for continuous variables. The cut points for high and low consumption were based on the restricted cubic spline analyses (see below and results).

Cox proportional hazard models with age as the underlying timescale were used to estimate hazard ratios (HR) between the intake of processed meat, red meat, the total intake of red and processed meat, lean and fatty fish, and mortality, and between the substitution of processed meat, of red meat, and of the total intake of red and processed meat with lean or fatty fish and mortality. The proportional hazards assumption was evaluated visually using log-log plots and Schoenfeld residuals.

The association between intake of processed meat, red meat, red and processed meat, lean and fatty fish, and mortality outcomes was investigated for non-linearity using restricted cubic splines with three knots placed at the 10th, 50th and 90th percentiles.

Specified substitution analysis was performed using the ‘Leave-one-out’ method to estimate the association between the replacement of 20 g/d of processed meat, 20 g/d of red meat and 20 g/d of red and processed meat with 20 g/d of lean or fatty fish^(29)^. The model for substitution of processed meat with lean or fatty fish can be parameterised as

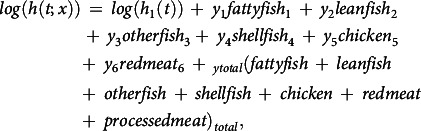

where the total variable is the sum of the intakes of processed meat, red meat, lean fish, fatty fish, and other foods in similar food groups, that is, other fish (including ‘other fish’, roe, caviar and liver), shellfish and chicken. When processed meat was not included and red meat was, the coefficient for lean or fatty fish represented the replacement of processed meat with lean or fatty fish, respectively.

We adjusted for various covariates in four different models. Model 1a was mutually adjusted for lean fish, fatty fish, red meat, processed meat, other fish, shellfish, and chicken, and additionally adjusted for age (continuous timescale), energy intake (continuous kJ/d (excluding energy from alcohol)), and for subcohorts (*n* 5), which was included as a stratum variable.

In model 1b, which is specified as our main model, we additionally adjusted for physical activity divided into three categories (low (≤ 4), moderate (5–6) or high (≥ 7)), smoking divided into six categories (never smokers, current heavy smokers, current moderate smokers, current smokers late starter, former smoker early starter and former smoker late starter) and alcohol intake divided into three categories (non-consumers, low consumers (0–5 g/d) and higher consumers (> 5 g/d)). In model 2, we further adjusted for the consumption of other food groups that are related to meat consumption and mortality, including fruits and vegetables, dairy products, wholegrain products, refined grain products and potatoes (all continuous in g/d). In model 3, we further adjusted for BMI category (< 20, 20–24·9, 25–29·9, ≥ 30 kg/m^2^) and diabetes (yes/no).

Stata/MP 16.0 was used to perform statistical analyses. Statistical significance was set at *P* < 0·05.

### Sensitivity analysis

The following two sensitivity analyses were conducted:

1. Because of concerns for reverse causation, we performed analyses starting at follow-up for all participants 2 years after enrolment.
2. Because of concerns due to missing data among covariates, we performed multiple imputation for the specified substitution analyses with processed meat and lean or fatty fish under the assumption that missing data could be missing at random. The imputation was performed by chained equations for missing data for the covariates: education (7–9, 10–12, 13–16 and ≥ 17 years of schooling), physical activity (continuous scale 1–10), smoking status (never smoker, current heavy smoker, current moderate smoker, current smoker late starter, former smoker early starter and former smoker late starter), height (cm) and weight (kg). The other covariates included in our models and mortality outcomes were included in the imputation models. The missing values were replaced with imputed values estimated based on observed values from twenty duplicated datasets. Imputed values were drawn with the use of predictive mean matching with the 100 nearest neighbours for physical activity, height and weight which were based on linear scales, and with the use of ordinal regression and multinominal regression to impute missing values for education and smoking, respectively.

## Results

We included 83 304 women in this study, of whom 9420 died during follow-up, including 4708 deaths from cancer and 1068 deaths from CVD (IHD or stroke) during a median follow-up time of 21·0 years (Table 1).

**Table 1.** Baseline characteristics for all women and for women with low and high intake of processed meat and for women with low and high intake of red meat

Characteristics	Cohort		Processed meat ≤ 30 g/d		Processed meat > 30 g/d		Red meat ≤ 20 g/d		Red meat > 20 g/d	
No. of participants *n*	83 304		39 119		42 255		55 476		22 121	
No. of total deaths	9420		4637		4515		6240		2482	
No. of deaths from cancer	4708		2227		2363		3080		1295	
No. of deaths from CVD	1068		550		491		716		275	

Age at baseline in years	Mean	sd	Mean	sd	Mean	sd	Mean	sd	Mean	sd
	51·6	6·4	52·5	6·5	50·8	6·1	51·8	6·6	51·2	5·9

Education*	*n*	%	*n*	%	*n*	%	*n*	%	*n*	%
7–9	19 873	23·9	9151	23·4	10 384	24·6	12 855	23·2	5934	26·8
10–12	28 984	34·8	13 023	33·3	15 452	36·6	18 978	34·2	8341	37·7
13–16	23 040	27·7	11 089	28·4	11 377	26·9	15 743	28·4	5562	25·1
≥ 17	11 407	13·7	5856	15·0	5042	11·9	7900	14·2	2284	10·3
Smoking*										
Never	29 684	35·6	14 359	36·7	14 592	34·5	20 890	37·7	6605	29·9
Current heavy smoker, early starter	5647	6·8	2300	5·9	3254	7·7	3248	5·9	2106	9·5
Current moderate smoker, early starter	10 816	13·0	4502	11·5	6173	14·6	6702	12·1	3561	16·1
Current smoker, late starter	7915	9·5	3710	9·5	4064	9·6	4977	9·0	2516	11·4
Former smoker, early starter	18 990	22·8	9027	23·1	9473	22·4	12 675	22·9	4887	22·1
Former smoker, late starter	10 252	12·3	5221	13·4	4699	11·1	6984	12·6	2446	11·1
Physical activity*										
Low	22 198	26·7	10 009	25·6	11 742	27·8	14 594	26·3	6206	28·1
Medium	36 028	43·3	16 788	42·9	18 500	43·8	24 160	43·6	9527	43·1
High	25 078	30·1	12 322	31·5	12 013	28·4	16 722	30·1	6388	28·9
Alcohol* (g/d)										
Non consumers	16 740	20·1	7776	19·9	8401	19·9	11 638	21·0	3384	15·3
0–5	45 895	55·1	21 600	55·2	23 379	55·3	31 066	56·0	11 953	54·0
> 5	20 669	24·8	9743	24·9	10 475	24·8	12 772	23·0	6784	30·7
BMI*										
< 20	5414	6·5	2525	6·5	2644	6·3	3495	6·3	1369	6·2
20–24·9	44 873	53·9	21 651	55·4	22 075	52·2	30 006	54·1	11 628	52·6
25–29·9	25 188	30·2	11 730	30·0	13 035	30·9	16 848	30·4	6882	31·1
≥ 30	7829	9·4	3213	8·2	4501	10·7	5127	9·2	2242	10·1
No. of participants with diabetes	1483	1·8	624	1·6	834	2·0	992	1·8	389	1·8

Dietary covariates	Median	Percentile 10–90	Median	Percentile 10–90	Median	Percentile 10–90	Median	Percentile 10–90	Median	Percentile 10–90
Energy intake (kJ/d)	6790	4686–9343	6289	4343–8623	7301	5238–9845	6678	4636–9142	7205	5050–9916
Processed meat (g/d)	30	9–62	18	8–27	45	32–73	28	10–59	38	15–71
Red meat (g/d)	13	3–28	11	2–25	16	5–32	11	4–17	26	21–41
Lean fish (g/d)	36	10–84	33	8–83	39	12–86	35	10–82	39	11–91
Fatty fish (g/d)	16	3–43	16	3–44	16	3–42	16	3–42	17	3–46
Fruits and vegetables (g/d)	304	125–598	310	125–614	295	124–572	300	124–586	307	129–603
Wholegrain products (g/d)	121	34–201	111	34–201	121	34–201	121	34–201	121	34–201
Refined grain products (g/d)	34	10–76	31	10–73	36	10–78	34	10–73	34	10–78
Potatoes (g/d)	126	22–189	126	22–189	126	22–189	126	22–189	126	22–189
Dairy products (g/d)	219	45–604	210	44–592	228	49–614	221	49–604	218	42–609

No, number of participants.

* Percent by columns.

### Test for linearity

**Fig. 2.**
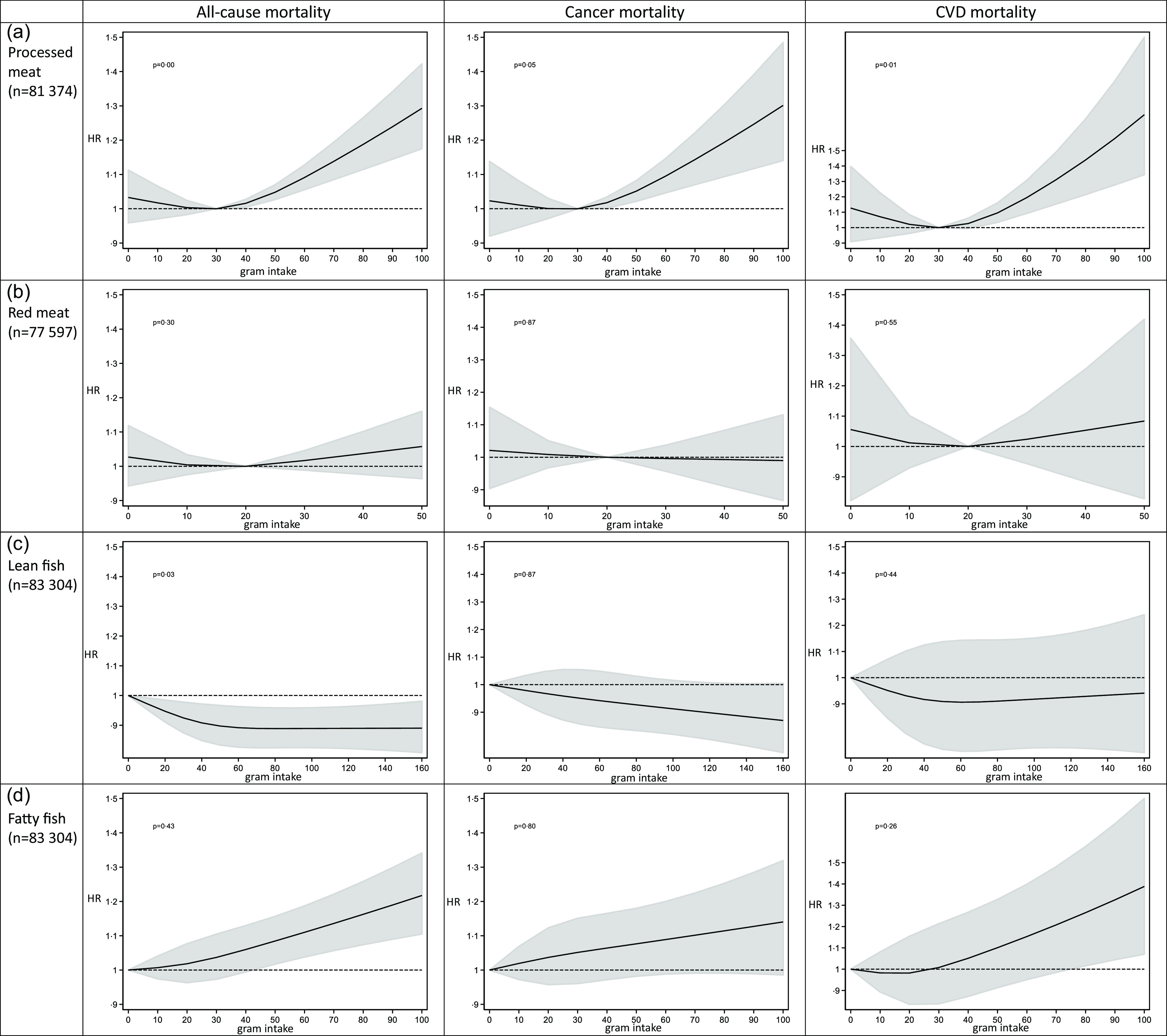
Intake of processed meat, red meat, lean and fatty fish and cause-specific mortality by restricted cubic spline regression.

The restricted cubic spline analyses showed that the association between the intake of processed meat and mortality was significantly non-linear, with the nadir of the curve around an intake of 30 g processed meat/d (Fig. 2(a)). The intake of red meat did not show a significant deviation from linearity in relation to mortality outcomes, but the level of intake that exhibited a non-significant trend towards the lowest all-cause and CVD mortality was approximately 20 g per d (Fig. 2(b)). Red and processed meat combined was significantly non-linearly associated with mortality outcomes, with the nadir of the curve around an intake of 50 g/d (online Supplementary Fig. 2). Based on these results, we decided to split the subsequent analyses between higher (> 30 g/d) and lower (≤ 30 g/d) intakes of processed meat, between higher (> 20 g/d) and lower (≤ 20 g/d) intakes of red meat and between higher (> 50 g/d) and lower intakes of red and processed meat (≤ 50 g/d).

The restricted cubic spline analysis estimating the association between lean fish consumption and all-cause mortality was non-linear with the curve being at its steepest between 0 g/d and approximately 40 g/d, before flattening out about 60 g/d (Fig. 2(c)). Since all intake levels of lean fish were beneficial, we treated it as a linear exposure in the following analyses. Fatty fish intake did not deviate from linearity in relation to mortality outcomes and was thus treated as a linear exposure in the following analyses (Fig. 2(d)).

### Baseline characteristics for high and low consumers of red and processed meat

Table 1 presents the baseline characteristics of all participants and the high and low processed meat consumers, and the high and low red meat consumers. We note that there were tendencies to a less health-conscious lifestyle among high consumers of processed meat, but also higher energy intake, and consequently higher intakes of most food groups including red meat and lean fish. They were also younger and had lower education than low consumers. There were similar tendencies, but weaker, among higher consumers of red meat.

### Red and processed meat and fish consumption in relation to mortality

Processed meat consumption was associated with higher all-cause, cancer and CVD mortality among women consuming > 30 g/d, while no significant association was observed between processed meat consumption and mortality outcomes among women consuming ≤ 30 g/d (Table 2). No significant associations between red meat consumption and mortality outcomes were observed either among high or low consumers of red meat (Table 2). Total consumption of red and processed meat was associated with higher all-cause, cancer and CVD mortality among women with higher red and processed meat intake (> 50 g/d), while no significant association was observed among women consuming ≤ 50 g of red and processed meat/d (Table 2). Lean fish consumption was marginally associated with lower all-cause and cancer mortality, while fatty fish consumption was marginally associated with higher all-cause and cancer mortality and with higher CVD mortality (Table 2).

**Table 2.** Hazard ratios (HR) and cause-specific mortality according to intake of processed meat, red meat, red and processed meat combined, lean and fatty fish

	All-cause mortality							
	Model 1a^††^		Model 1b^‡‡^		Model 2^§§^		Model 3^||||^	
Per 20 g/d	HR	95 % CI	HR	95 % CI	HR	95 % CI	HR	95 % CI
Processed meat > 30 g per d^*^	1·12	1·09, 1·16	1·07	1·04, 1·11	1·07	1·04, 1·11	1·06	1·03, 1·10
Processed meat ≤ 30 g per d^†^	1·00	0·92, 1·08	0·97	0·89, 1·05	0·98	0·90, 1·06	0·97	0·90, 1·06
Red meat > 20 g per d^‡^	1·16	1·07, 1·26	1·06	0·97, 1·14	1·06	0·98, 1·15	1·06	0·97, 1·15
Red meat ≤ 20 g per d^§^	1·07	0·97, 1·19	1·00	0·90, 1·11	1·00	0·90, 1·11	1·01	0·91, 1·12
Red and processed meat > 50 g per d^||^	1·12	1·08, 1·15	1·06	1·03, 1·09	1·06	1·02, 1·09	1·05	1·02, 1·08
Red and processed meat ≤ 50 g per d^¶^	1·03	0·98, 1·08	0·98	0·94, 1·03	0·99	0·95, 1·04	0·99	0·95, 1·04
Lean fish^**^	0·99	0·98, 1·01	0·99	0·97, 1·00	0·99	0·98, 1·00	0·99	0·98, 1·00
Fatty fish^**^	1·04	1·02, 1·06	1·03	1·01, 1·06	1·04	1·02, 1·06	1·04	1·02, 1·06

	Cancer mortality							
	Model 1a		Model 1b		Model 2		Model 3	
	HR	95 % CI	HR	95 % CI	HR	95 % CI	HR	95 % CI
Processed meat > 30 g per d^*^	1·12	1·07, 1·17	1·08	1·03, 1·13	1·08	1·04, 1·13	1·08	1·04, 1·13
Processed meat ≤ 30 g per d^†^	0·96	0·86, 1·08	0·93	0·83, 1·04	0·93	0·83, 1·05	0·93	0·83, 1·05
Red meat > 20 g per d^‡^	1·05	0·94, 1·18	0·97	0·86, 1·09	0·97	0·86, 1·09	0·97	0·86, 1·09
Red meat ≤ 20 g per d^§^	1·04	0·89, 1·20	0·97	0·83, 1·13	0·97	0·84, 1·13	0·98	0·84, 1·14
Red and processed meat > 50 g per d^||^	1·09	1·05, 1·13	1·04	1·00, 1·08	1·05	1·00, 1·09	1·04	1·00, 1·09
Red and processed meat ≤ 50 g per d^¶^	1·00	0·94, 1·07	0·96	0·90, 1·02	0·96	0·90, 1·03	0·96	0·90, 1·03
Lean fish^**^	0·99	0·97, 1·01	0·98	0·97, 1·00	0·98	0·96, 1·00	0·98	0·97, 1·00
Fatty fish^**^	1·04	1·01, 1·07	1·03	1·00, 1·06	1·03	1·00, 1·06	1·03	1·00, 1·06

	CVD mortality							
	Model 1a		Model 1b		Model 2		Model 3	
	HR	95 % CI	HR	95 % CI	HR	95 % CI	HR	95 % CI
Processed meat > 30 g per d^*^	1·26	1·16, 1·37	1·19	1·09, 1·30	1·20	1·10, 1·31	1·16	1·06, 1·27
Processed meat ≤ 30 g per d^†^	1·09	0·87, 1·38	1·06	0·84, 1·34	1·09	0·86, 1·38	1·06	0·83, 1·34
Red meat > 20 g per d^‡^	1·23	0·98, 1·55	1·09	0·86, 1·37	1·10	0·87, 1·39	1·10	0·87, 1·39
Red meat ≤ 20 g per d^§^	1·03	0·76, 1·40	0·94	0·69, 1·29	0·95	0·70, 1·30	0·95	0·69, 1·29
Red and processed meat > 50 g per d^||^	1·22	1·13, 1·32	1·14	1·05, 1·23	1·14	1·05, 1·23	1·12	1·03, 1·21
Red and processed meat ≤ 50 g per d^¶^	1·07	0·93, 1·22	1·01	0·88, 1·15	1·04	0·91, 1·19	1·02	0·89, 1·17
Lean fish^**^	1·01	0·98, 1·05	0·99	0·96, 1·03	1·00	0·97, 1·04	1·00	0·96, 1·03
Fatty fish^**^	1·08	1·02, 1·14	1·07	1·02, 1·13	1·07	1·02, 1·13	1·06	1·01, 1·12

* *n* 42 255, no. of deaths = 4515, no. of cancer-related deaths = 2363, no. of CVD-related deaths = 491.

† *n* 39 119, no. of deaths = 4637, no. of cancer-related deaths = 2227, no. of CVD-related deaths = 550.

‡ *n* 22 121, no. of deaths = 2482, no. of cancer-related deaths = 1295, no. of CVD-related deaths = 275.

§ *n* 55 476, no. of deaths = 6240, no. of cancer-related deaths = 3080, no. of CVD-related deaths = 716.

|| *n* 34 959, no. of deaths = 3784, no. of cancer-related deaths = 2002, no. of CVD-related deaths = 420.

¶ *n* 47 286, no. of deaths = 5501, no. of cancer-related deaths = 2645, no. of CVD-related deaths = 635.

** *n* 83 304, no. of deaths = 9420, no. of cancer-related deaths = 4708, no. of CVD-related deaths = 1068.

†† Mutually adjusted for red meat, processed meat, lean fish, fatty fish, chicken, other fish, shellfish (with the exposure omitted in the respective analyses), age (underlying timescale) and energy intake (continuous kJ/d excluding energy from alcohol), stratified by subcohorts (*n* 5).

‡‡ Model 1a + adjusted for education (7–9, 10–12, 13–16 and ≥ 17 years of schooling), alcohol (non-consumer, 0–5, > 5 g/d), smoking (never, current heavy smoker, current moderate smoker, current smoker late starter, former smoker early starter, former smoker late starter) and physical activity (low, medium, high).

§§ Model 1b + adjusted for other foods: fruits and vegetables, wholegrain products, refined grain products, potatoes, dairy products (g/d continuous).

|||| Model 2 + adjusted for BMI categories (< 20, 20–24·99, 25–29·99, > 30), diabetes (yes/no).

### Specified substitution analyses

Replacing 20 g processed meat/d with 20 g lean fish was associated with 8% lower all-cause mortality (HR 0·92, 95% CI 0·89, 0·96), 8 % lower cancer mortality (HR 0·92, 95% CI 0·88, 0·97) and 18 % lower CVD mortality (HR 0·82, 95% CI 0·74, 0·90) among women consuming > 30 g processed meat/d (Table 3). Replacing 20 g processed meat/d with 20 g fatty fish was among high consumers of processed meat associated with 13 % lower CVD mortality (HR 0·87, 95% CI 0·77, 0·97), but not statistically significantly with all-cause mortality (HR 0·97, 95% CI 0·93, 1·01) or cancer mortality (HR 0·96, 95% CI 0·90, 1·01) (Table 3). Replacing processed meat with lean or fatty fish was not significantly associated with mortality outcomes among lower consumers of processed meat ≤ 30 g/d (Table 3).

**Table 3.** Hazard ratios (HR) and cause-specific mortality according to specified substitution analyses of processed meat with lean or fatty fish for women consuming > 30 g and ≤ 30 g processed meat per d

3a. Specified substitution analyses for processed meat intake > 30 g per d								
	All-cause mortality (no. of deaths = 4515)							
*n* 42 255	Model 1a^*^		Model 1b^†^		Model 2^‡^		Model 3^§^	
Per 20 g/d	HR	95 % CI	HR	95 % CI	HR	95 % CI	HR	95 % CI
Lean fish for processed meat	0·89	0·86, 0·92	0·92	0·89, 0·96	0·92	0·89, 0·96	0·93	0·90, 0·97
Fatty fish for processed meat	0·93	0·89, 0·97	0·97	0·93, 1·01	0·97	0·93, 1·01	0·97	0·93, 1·01

	Cancer mortality (no. of deaths = 2363)							
Per 20 g/d	Model 1a^*^		Model 1b^†^		Model 2^‡^		Model 3^§^	
	HR	95 % CI	HR	95 % CI	HR	95 % CI	HR	95 % CI
Lean fish for processed meat	0·90	0·85, 0·94	0·92	0·88, 0·97	0·92	0·87, 0·96	0·92	0·87, 0·97
Fatty fish for processed meat	0·93	0·88, 0·98	0·96	0·90, 1·01	0·95	0·90, 1·01	0·95	0·90, 1·01

	CVD mortality (no. of deaths = 491)							
Per 20 g/d	Model 1a^*^		Model 1b^†^		Model 2^‡^		Model 3^§^	
	HR	95 % CI	HR	95 % CI	HR	95 % CI	HR	95 % CI
Lean fish for processed meat	0·79	0·72, 0·87	0·82	0·74, 0·90	0·82	0·74, 0·91	0·84	0·76, 0·93
Fatty fish for processed meat	0·82	0·73, 0·92	0·87	0·77, 0·97	0·87	0·77, 0·97	0·89	0·79, 1·00

3b. Specified substitution analyses for processed meat intake ≤ 30 g per d								
	All-cause mortality (no. of deaths = 4637)							
*n* 39 119	Model 1a^*^		Model 1b^†^		Model 2^‡^		Model 3^§^	
Per 20 g/d	HR	95 % CI	HR	95 % CI	HR	95 % CI	HR	95 % CI
Lean fish for processed meat	0·99	0·91, 1·08	1·01	0·93, 1·10	1·01	0·93, 1·10	1·02	0·93, 1·10
Fatty fish for processed meat	1·05	0·96, 1·14	1·07	0·98, 1·16	1·07	0·98, 1·17	1·07	0·98, 1·17

	Cancer mortality (no. of deaths = 2227)							
Per 20 g/d	Model 1a^*^		Model 1b^†^		Model 2^‡^		Model 3^§^	
	HR	95 % CI	HR	95 % CI	HR	95 % CI	HR	95 % CI
Lean fish for processed meat	1·02	0·90, 1·14	1·05	0·93, 1·18	1·04	0·93, 1·18	1·04	0·92, 1·18
Fatty fish for processed meat	1·07	0·94, 1·20	1·10	0·97, 1·24	1·10	0·98, 1·25	1·10	0·98, 1·25

	CVD mortality (no. of deaths = 550)							
Per 20 g/d	Model 1a^*^		Model 1b^†^		Model 2^‡^		Model 3^§^	
	HR	95 % CI	HR	95 % CI	HR	95 % CI	HR	95 % CI
Lean fish for processed meat	0·93	0·73, 1·18	0·95	0·75, 1·20	0·93	0·74, 1·19	0·96	0·75, 1·22
Fatty fish for processed meat	1·01	0·79, 1·28	1·02	0·80, 1·30	0·99	0·78, 1·27	1·02	0·80, 1·31

* Mutually adjusted for red meat, lean fish, fatty fish, chicken, other fish, shellfish, age (underlying timescale) and energy intake (continuous kJ/d excluding energy from alcohol), stratified by subcohorts (*n* 5).

† Model 1a + adjusted for education (7–9, 10–12, 13–16, ≥ 17 years of schooling), alcohol (non-consumer, 0–5, > 5 g/d), smoking (never, current heavy smoker, current moderate smoker, current smoker late starter, former smoker early starter, former smoker late starter) and physical activity (low, medium, high).

‡ Model 1b + adjusted for other foods: fruits and vegetables, wholegrain products, refined grain products, potatoes, and dairy products (g/d continuous).

§ Model 2 + adjusted for BMI categories (< 20, 20–24·99, 25–29·99, > 30) and diabetes (yes/no).

Replacing 20 g of red meat/d with 20 g of lean fish was among women consuming > 20 g red meat/d not statistically significantly associated with all-cause mortality (HR 0·93, 95 % CI 0·86, 1·01), cancer mortality (model 1b: HR 1·03, 95 % CI 0·92, 1·17) or CVD mortality (HR 0·88, 95 % CI 0·69, 1·12) (Table 4). Among higher red meat consumers (> 20 g/d), replacing red meat with fatty fish was not significantly associated with all-cause mortality (HR 0·99, 95 % CI 0·91, 1·08), cancer mortality (HR 1·06, 95 % CI 0·93, 1·21) or CVD mortality (HR 1·00, 95 % CI 0·77, 1·29) (Table 4). No associations were observed between replacement of red meat with fish among women consuming ≤ 20 g of red meat/d (Table 4).

**Table 4.** Hazard ratios (HR) and cause-specific mortality according to specified substitution analyses of red meat with lean or fatty fish for women consuming > 20 g and ≤ 20 g red meat per d

4a. Specified substitution analyses for red meat intake > 20 g per d								
	All-cause mortality (no. of deaths = 2482)							
*n* 22 121	Model 1a^*^		Model 1b^†^		Model 2^‡^		Model 3^§^	
Per 20 g/d	HR	95 % CI	HR	95 % CI	HR	95 % CI	HR	95 % CI
Lean fish for red meat	0·86	0·79, 0·93	0·93	0·86, 1·01	0·93	0·86, 1·01	0·93	0·86, 1·02
Fatty fish for red meat	0·90	0·83, 0·98	0·99	0·91, 1·08	0·99	0·91, 1·08	0·99	0·91, 1·08

	Cancer mortality (no. of deaths = 1295)							
Per 20 g/d	Model 1a^*^		Model 1b^†^		Model 2^‡^		Model 3^§^	
	HR	95 % CI	HR	95 % CI	HR	95 % CI	HR	95 % CI
Lean fish for red meat	0·96	0·85, 1·08	1·03	0·92, 1·17	1·03	0·92, 1·16	1·03	0·91, 1·16
Fatty fish for red meat	0·98	0·86, 1·11	1·06	0·93, 1·21	1·06	0·93, 1·21	1·06	0·93, 1·21

	CVD mortality (no. of deaths = 275)							
Per 20 g/d	Model 1a^*^		Model 1b^†^		Model 2^‡^		Model 3^§^	
	HR	95 % CI	HR	95 % CI	HR	95 % CI	HR	95 % CI
Lean fish for red meat	0·80	0·63, 1·01	0·88	0·69, 1·12	0·88	0·69, 1·12	0·88	0·69, 1·12
Fatty fish for red meat	0·90	0·67, 1·12	1·00	0·77, 1·29	1·00	0·77, 1·29	0·98	0·76, 1·27

4b. Specified substitution analyses for red meat intake ≤ 20 g per d								
	All-cause mortality (no. of deaths = 6240)							
*n* 55 476	Model 1a^*^		Model 1b^†^		Model 2^‡^		Model 3^§^	
Per 20 g/d	HR	95 % CI	HR	95 % CI	HR	95 % CI	HR	95 % CI
Lean fish for red meat	0·93	0·83, 1·03	0·99	0·89, 1·10	0·99	0·89, 1·10	0·98	0·88, 1·09
Fatty fish for red meat	0·97	0·87, 1·08	1·04	0·93, 1·15	1·04	0·93, 1·15	1·02	0·92, 1·14

	Cancer mortality (no. of deaths = 3080)							
Per 20 g/d	Model 1a^*^		Model 1b^†^		Model 2^‡^		Model 3^§^	
	HR	95 % CI	HR	95 % CI	HR	95 % CI	HR	95 % CI
Lean fish for red meat	0·95	0·82, 1·11	1·01	0·87, 1·17	1·01	0·86, 1·17	1·00	0·86, 1·16
Fatty fish for red meat	1·00	0·86, 1·17	1·07	0·91, 1·24	1·06	0·91, 1·24	1·06	0·91, 1·23

	CVD mortality (no. of deaths = 716)							
Per 20 g/d	Model 1a^*^		Model 1b^†^		Model 2^‡^		Model 3^§^	
	HR	95 % CI	HR	95 % CI	HR	95 % CI	HR	95 % CI
Lean fish for red meat	0·98	0·72, 1·35	1·06	0·78, 1·45	1·06	0·77, 1·45	1·06	0·77, 1·45
Fatty fish for red meat	1·03	0·75, 1·41	1·11	0·81, 1·53	1·10	0·80, 1·51	1·10	0·80, 1·52

* Mutually adjusted for processed meat, lean fish, fatty fish, chicken, other fish, shellfish, age (underlying timescale) and energy intake (continuous kJ/d excluding energy from alcohol), stratified by subcohorts (*n* 5).

† Model 1a + adjusted for education (7–9, 10–12, 13–16, ≥ 17 years of schooling), alcohol (non-consumer, 0–5, > 5 g/d), smoking (never, current heavy smoker, current moderate smoker, current smoker late starter, former smoker early starter, former smoker late starter) and physical activity (low, medium, high).

‡ Model 1b + adjusted for other foods: fruits and vegetables, wholegrain products, refined grain products, potatoes, and dairy products (g/d continuous).

§ Model 2 + adjusted for BMI categories (< 20, 20–24·99, 25–29·99, > 30) and diabetes (yes/no).

Overall, additional adjustments for other foods (model 2) and potential mediators BMI and diabetes (model 3) did not lead to significant changes in any of the presented associations (Table 2–4).

For the specified substitution analyses replacing red and processed meat with lean fish, we observed lower all-cause and CVD mortality, but not cancer mortality, among women consuming > 50 g of red and processed meat/d (online Supplementary Table 1(a)). No associations with mortality were observed with replacing red and processed meat with lean fish among low consumers of red and processed meat (online Supplementary Table 1(b)). Replacing red and processed meat with fatty fish was not associated with mortality outcomes among high consumers of red and processed meat (online Supplementary Table 1(a)), while higher all-cause and cancer mortality was observed with replacing red and processed meat with fatty fish among low consumers of red and processed meat (online Supplementary Table 1(b)).

### Sensitivity analyses

Starting follow-up for all participants 2 years after enrolment did not change our main results (online Supplementary Table 2–3).

Conducting multiple imputation for handling missing data among covariates gave similar results as our complete-case analyses (online Supplementary Table 4).

## Discussion

In this prospective cohort study of Norwegian women, we observed non-linear associations between processed meat and red and processed meat consumption and mortality which led to separate analyses for high and low consumers of meat. We observed that higher consumption of processed meat can increase the risk of premature death including death from cancer and IHD and stroke, while this risk was not evident at lower consumption levels of processed meat. Red meat consumption was not significantly associated with mortality even at higher intake levels. Expanding our analyses to the combined intake of red and processed meat revealed similar associations as with processed meat. Higher intake of lean fish was beneficial, while higher fatty fish intake was associated with higher all-cause and CVD mortality. Among women with higher processed meat intake (> 30 g/d), replacing processed meat with lean fish was associated with 8% lower all-cause mortality and cancer mortality and with 18 % lower CVD mortality (per 20 g/d replacement). Replacement of processed meat with fatty fish among higher processed meat consumers was associated with 13 % lower CVD mortality per 20 g/d replacement. No associations were observed in women with lower processed meat intake. Replacing red meat with lean or fatty fish was not significantly associated with mortality outcomes. When the substitution analyses were expanded to the combined intake of red and processed meat, only substitution with lean fish was beneficial among high consumers, while among low consumers we observed higher all-cause and cancer mortality when replaced with fatty fish.

### Explanation of findings

The stronger associations between processed meat intake compared with red meat intake and mortality in high consumers of meat are probably due to different nutritional composition and preparation methods of red and processed meat. Processed meat usually has higher energy density and lower levels of essential nutrients typically present in red meat as well as higher levels of Na and additives. The observed differences in mortality by replacing processed meat with fish in different strata of processed meat intake may be attributed to that the incorporation of processed meat in the diet enhances dietary diversity and provides essential nutrients like Fe. Alternatively, it is plausible that adverse health effects from processed meat primarily manifest when the intake of some nutrients and substances reaches a threshold, and thus that replacing lower intake levels of processed meat with fish has less impact. Moreover, a higher consumption of processed meat tends to displace other food items, resulting in reduced dietary variety. Lower intake of SFA or the replacement of SFA with unsaturated fatty acids may play a significant role in the strongest association observed between the substitution of processed meat with lean or fatty fish in relation to CVD mortality in high processed meat consumers^(30,31)^. The observed linear association between higher intake of fatty fish and higher all-cause mortality is somewhat different from our previous analyses on fatty fish and all-cause mortality in the NOWAC cohort where we observed a J-shaped curve^(15)^. This might be explained by the inclusion of processed fish such as mackerel in tomato which contains added sugar, Na and preservatives, in current analyses.

### Findings from other studies

To the best of our knowledge, no previous studies are directly comparable to the present one, as they have not examined the association between replacing red and/or processed meat with lean or fatty fish, while stratifying the analyses based on intake level of red and processed meat. Nevertheless, a few previous studies have assessed the association between replacing red and processed meat with fish and mortality. None of these studies were, however, restricted to women, nor did they present sex-specific results. Nielsen et al. found similar results as we did in The Danish Diet, Cancer and Health cohort study, which is quite comparable to our cohort study, both in terms of geographical proximity and food culture^(11)^. Their findings indicated that replacing processed meat with fish or poultry showed a stronger association with lower mortality compared with replacing red meat. Specifically, they observed that 150 g of processed meat/week (which is comparable to 20 g/d used in present study), with total fish, was associated with lower all-cause and cancer mortality, but not with CVD mortality, in men and women. Deviating results on CVD mortality between our studies might be explained by the different definitions of CVD-related deaths, as Nielsen et al. included ICD-10 codes I00-I99, while we only included IHD and stroke, which are the leading causes of CVD-related deaths. In line with our results, Pan et al. found that replacing one serving of processed meat per d (85 g/d) with one serving of fish was associated with 10 % lower all-cause mortality in a cohort of men and women from the USA^(13)^. In contrast to our results, they found that red and processed meat intake was linearly associated with higher mortality, and that substituting red meat with fish was associated with lower mortality, although to a lesser extent than the association observed with processed meat^(19)^. In another study from the USA, Etemadi et al. found that intake of both red and processed meat was associated with higher mortality, and that 20 g per 1000 kcal increased daily intake of fish and similarly decreased intake of red and processed meat was associated with 5% lower all-cause mortality in men and women^(14)^. One more study including US men and women by Zhong *et al.* found that substituting both red meat and processed meat with fish could reduce all-cause mortality^(12)^. In line with our results, van den Brandt *et al.* observed that processed meat intake was associated with overall higher mortality in men and women, while red meat intake was not^(32)^. However, they found no deviation from linearity between processed consumption and all-cause, cancer or CVD mortality. They observed higher all-cause and cause-specific mortality from higher fish consumption, and that replacing processed meat with fish was not significantly associated with all-cause, cancer or CVD mortality, but the HR was elevated for all outcomes. One might consider whether consuming fish like salmon and herring of possible Baltic Sea origin which exhibits higher levels of dioxins and polychlorinated biphenyls (PCBs) than fishes of non-Baltic origin could potentially have undermined the benefits of replacing processed meat with fish in the Dutch study^(33)^. However, these are mere speculations since the origin of the fish consumed is unknown.

### Strengths and limitations

These findings should be interpreted with caution, as the lower mortality observed from replacing processed meat and red and processed meat combined with particularly lean fish is limited to interpretation using statistical methods and is not based on an observed effect from actual dietary changes. However, intervention studies are poorly suited for investigating dietary interventions and outcomes that require a long follow-up period, such as mortality. The strength of this study was that it included a nationally representative cohort of women with a low risk of sampling bias and high external validity. The linkage to the death registry of Norway, which confirms all deaths, lowers the risk of misclassification, although the cause of death may be misclassified. The large sample size and long follow-up time provided a high number of deaths, strengthening the statistical power in the analyses and making it possible to perform analyses in subsamples of the study sample. Furthermore, validated FFQ with detailed information on different types of fish facilitated a good measure of lean and fatty fish exposure and allowed for separate analyses of lean and fatty fish. However, the study was limited by self-reported dietary intake, which is prone to error and unlikely to be precise. The meat consumption, as estimated through four repeated 24-h dietary recalls in a validation study, was however not significantly different from the amount estimated using the FFQ. Conversely, the intake of fish, as estimated in the FFQ, was higher than the estimations derived from the 24-h dietary recalls^(22)^. The actual consumption of meats and fish is nevertheless underestimated due to the unknown amount from combined dishes. In the validation study, combined dishes were treated as grams of the dish and not as grams of its ingredients. Another limitation is that we were unable to capture changes in diet or covariates over time, as we only used one time point for exposure measurements.

Errors due to self-reporting of covariates and residual confounding from unmeasured factors can introduce bias. Hence, we cannot rule out that the beneficial effect on mortality from replacing processed meat with fish can be attributed to lifestyle factors associated with fish consumption or high consumption of processed meat, or with other foods often consumed together with these protein sources. For example, the composition of meals with fish compared with processed meat might be healthier in general. This has been shown in a study comparing nutritional composition between red meat dinners (including processed meat) and fish dinners in Norwegian adults where fish dinners generally had a healthier profile with less energy and a higher percentage of energy from proteins than red meat dinners^(34)^. Adjusting for other foods in our analyses did, however, not change the association between replacing processed meat and red meat with lean or fatty fish. In a previous study on dietary patterns in NOWAC, fish eaters were characterised by a high intake of fat and boiled coffee, current smoking, lower education, and higher BMI than women belonging to different dietary clusters, indicating a less healthy lifestyle among fish eaters^(35)^. These characteristics may however not accurately reflect the diverse range of dietary habits and lifestyles among all fish eaters, as fish consumption has been associated with overall healthier meal compositions and lifestyles^(34,36)^. It is also likely that there may be some residual confounding by smoking, a major predictor of mortality, in the analysis. The relatively high number of participants with missing data for included covariates could bias the observed associations. However, the fact that our main results for substitution of processed meat with lean or fatty fish were similar after imputing missing values suggests that the observed associations from the complete-case analyses is quite robust. Furthermore, we chose to do substitution by weight, rather than by energy, and the difference in energy content between red and processed meat and particularly with lean fish leaves an unspecified energy substitution that must be replaced by other foods that were not controlled in the analyses.

### Public health implications

The findings of this study align with the Nordic Nutrition Recommendations 2023, which suggest limiting the consumption of red and processed meat to a maximum of 350 g per week for health purposes, as we observed that an intake above this was associated with higher mortality^(1)^. However, our results emphasise the significant role of processed meat in explaining the positive association between red and processed meat consumption and mortality.

The potential reduction of premature deaths in high processed meat consumers by replacing some of the processed meat intake with particularly lean fish could be substantial in a public health perspective as the estimated intake of processed meat among women in Norway is higher than recommended^(1,37)^. The replacement of processed meat with fish of equal serving size is applicable to traditional Norwegian meal settings and can provide an easy interpretation from a public health perspective. Implementing such a transition is however not straightforward, and a study conducted by Erkkola *et al.* in Finland highlighted that when individuals make transitions away from red meat consumption, they tend to shift their dietary preferences towards poultry over fish^(38)^.

### Conclusion

Our study indicates that higher consumption of processed meat, but not red meat, is associated with higher cause-specific mortality, while lower processed meat consumption may not increase the risk of premature death among women in Norway. While lean fish consumption was associated with lower all-cause mortality, higher consumption of fatty fish was associated with higher all-cause and CVD mortality.

Replacing processed meat with lean fish in higher processed meat consumers could potentially lower the risk of premature deaths from all causes, including cancer and CVD in Norwegian women. Replacing processed meat intake with fatty fish may specifically reduce the risk of early death from IHD and stroke in women with higher processed meat consumption. It is important to highlight that our observations regarding benefits of replacing processed meat with fish were restricted to women with higher processed meat consumption. Further investigation is warranted to confirm these results and to understand the potential effects of replacements of processed meat with lean and fatty fish in women with lower processed meat intake and in men.

## Supplementary material

For supplementary material accompanying this paper visit https://doi.org/10.1017/S0007114523002040. click here to view supplementary material
