# Supplementary material for: Replacing red and processed meat with lean or fatty fish and all-cause and cause-specific mortality in Norwegian women. The Norwegian Women and Cancer Study (NOWAC): a prospective cohort study
Source: Br J Nutr. 2023 Sep 11;131(3):531–43. doi: 10.1017/S0007114523002040 (PMC10784130; doi:10.1017/S0007114523002040)
Supplement: Supplementary file 1 [file S0007114523002040sup001.docx]

Contents

[1](#_Toc141954555)

[Supplemental Tables 2](#_Toc141954556)

[Supplemental table 1. Hazard ratio (HR) and cause specific mortality according to specified substitution analyses for red and processed meat with lean or fatty fish for women consuming >50 grams and ≤ 50 grams red and processed meat 2](#_Toc141954557)

[Supplemental Table 2. Hazard ratios (HR) and cause specific mortality according to specified substitution analyses of processed meat with lean or fatty fish for women consuming >30 grams and ≤ 30 grams processed meat starting follow-up three years after baseline 3](#_Toc141954558)

[Supplemental Table 3. Hazard ratios (HR) and cause specific mortality according to specified substitution analyses of red meat with lean or fatty fish for women consuming >20 grams and ≤ 20 grams of red meat starting follow-up three years after baseline 4](#_Toc141954559)

[Supplemental Table 4. Hazard ratios (HR) and cause specific mortality according to specified substitution analyses of processed meat with lean or fatty fish for women consuming >30 grams and ≤ 30 grams processed meat per day using multiple imputation for missing values on confounding covariates 5](#_Toc141954560)

[Supplemental Figures 6](#_Toc141954561)

[Supplemental Figure 1. Directed acyclic graph (DAG) illustrating the hypothesized causal relationships between covariates in the association between the substitution of red and processed meat with lean or fatty fish and mortality 6](#_Toc141954562)

[Supplemental Figure 2. Intake of red and processed meat and cause specific mortality by restricted cubic spline regression 7](#_Toc141954563)

# Supplemental Tables

Supplemental table 1. Hazard ratio (HR) and cause specific mortality according to specified substitution analyses for red and processed meat with lean or fatty fish for women consuming >50 grams/day and ≤ 50 grams/day of red and processed meat

| ST1a. Specified substitution analyses for red and processed meat intake > 50 grams/day | | | | | | | | |
| --- | --- | --- | --- | --- | --- | --- | --- | --- |
| n= 34 959 | All-cause mortality (No. of deaths = 3 784) | | | | | | | |
| Per 20 g/day | Model 1a* | | Model 1b† | | Model 2‡ | | Model 3§ | |
|  | HR | 95% CI | HR | 95% CI | HR | 95% CI | HR | 95% CI |
| Lean fish for red and processed meat | 0.90 | 0.87-0.93 | 0.94 | 0.91-0.97 | 0.94 | 0.91-0.97 | 0.95 | 0.92-0.98 |
| Fatty fish for red and processed meat | 0.93 | 0.89-0.97 | 0.97 | 0.93-1.01 | 0.97 | 0.93-1.02 | 0.98 | 0.94-1.02 |
|  | Cancer mortality (No. of deaths = 2 002) | | | | | | | |
| Per 20 g/day | Model 1a* | | Model 1b† | | Model 2‡ | | Model 3§ | |
|  | HR | 95% CI | HR | 95% CI | HR | 95% CI | HR | 95% CI |
| Lean fish for red and processed meat | 0.93 | 0.88-0.97 | 0.96 | 0.92-1.01 | 0.96 | 0.91-1.01 | 0.96 | 0.91-1.01 |
| Fatty fish for red and processed meat | 0.94 | 0.88-1.00 | 0.97 | 0.92-1.03 | 0.97 | 0.91-1.03 | 0.97 | 0.91-1.03 |
|  | CVD mortality (No. of deaths = 420) | | | | | | | |
| Per 20 g/day | Model 1a* | | Model 1b† | | Model 2‡ | | Model 3§ | |
|  | HR | 95% CI | HR | 95% CI | HR | 95% CI | HR | 95% CI |
| Lean fish for red and processed meat | 0.81 | 0.73-0.89 | 0.85 | 0.77-0.93 | 0.85 | 0.77-0.94 | 0.86 | 0.78-0.95 |
| Fatty fish for red and processed meat | 0.86 | 0.77-0.97 | 0.92 | 0.82-1.04 | 0.93 | 0.82-1.04 | 0.93 | 0.83-1.05 |
| ST1b. Specified substitution analyses for red and processed meat intake ≤ 50 grams/day | | | | | | | | |
| n= 47 286 | All-cause mortality (No. of deaths = 5 501) | | | | | | | |
| Per 20 g/day | Model 1a* | | Model 1b† | | Model 2‡ | | Model 3§ | |
|  | HR | 95% CI | HR | 95% CI | HR | 95% CI | HR | 95% CI |
| Lean fish for red and processed meat | 0.96 | 0.91-1.01 | 1.00 | 0.94-1.05 | 0.99 | 0.95-1.04 | 0.99 | 0.95-1.04 |
| Fatty fish for red and processed meat | 1.02 | 0.97-1.07 | 1.07 | 1.01-1.12 | 1.06 | 1.00-1.11 | 1.06 | 1.00-1.11 |
|  | Cancer mortality (No. of deaths = 2 645) | | | | | | | |
| Per 20 g/day | Model 1a* | | Model 1b† | | Model 2‡ | | Model 3§ | |
|  | HR | 95% CI | HR | 95% CI | HR | 95% CI | HR | 95% CI |
| Lean fish for red and processed meat | 0.97 | 0.91-1.04 | 1.01 | 0.94-1.09 | 1.00 | 0.93-1.08 | 1.00 | 0.93-1.08 |
| Fatty fish for red and processed meat | 1.03 | 0.96-1.11 | 1.08 | 1.00-1.16 | 1.08 | 1.00-1.16 | 1.07 | 1.00-1.16 |
|  | CVD mortality (No. of deaths = 635) | | | | | | | |
| Per 20 g/day | Model 1a* | | Model 1b† | | Model 2‡ | | Model 3§ | |
|  | HR | 95% CI | HR | 95% CI | HR | 95% CI | HR | 95% CI |
| Lean fish for red and processed meat | 0.96 | 0.83-1.11 | 1.00 | 0.87-1.16 | 0.98 | 0.85-1.14 | 1.00 | 0.86-1.15 |
| Fatty fish for red and processed meat | 1.01 | 0.87-1.17 | 1.06 | 0.91-1.23 | 1.03 | 0.89-1.20 | 1.04 | 0.90-1.21 |

HR, hazard ratio

CI, confidence interval

CVD, cardiovascular disease

* Mutually adjusted for lean and fatty fish, chicken, other fish, shellfish, age (underlying timescale), energy intake (continuous kJ/day excluding energy from alcohol), stratified by subcohorts (n=5)

† model 1a + adjusted for education (7-9, 10-12, 13-16, ≥ 17 years of schooling), alcohol (non-consumer, 0–5,>5 g/day), smoking (never, current heavy smoker early starter, current moderate smoker early starter, current smoker late starter, former smoker early starter, former smoker late starter), physical activity (low, medium, high)

‡ model 1b + adjusted for other foods (fruits and vegetables, whole grain products, refined grains, potatoes, dairy products (grams/day continuous))

§ model 2 + adjusted for BMI categories (<20, 20-24.99,25-29.99, >30), type 2 diabetes

Supplemental Table 2. Hazard ratios (HR) and cause specific mortality according to specified substitution analyses of processed meat with lean or fatty fish for women consuming >30 grams/day and ≤ 30 grams/day of processed meat starting follow-up two years after baseline

| ST2a. Specified substitution analyses for processed meat intake > 30 grams/day | | | | | | |
| --- | --- | --- | --- | --- | --- | --- |
| n= 42 076 | All-cause  (No. of deaths = 4 350) | | Cancer  (No. of deaths = 2 265) | | CVD  (No. of deaths = 468) | |
| Per 20 g/day | HR | 95% CI | HR | 95% CI | HR | 95% CI |
| Lean fish for processed meat | 0.92 | 0.89-0.96 | 0.92 | 0.88-0.97 | 0.81 | 0.73-0.89 |
| Fatty fish for processed meat | 0.97 | 0.93-1.01 | 0.96 | 0.90-1.02 | 0.86 | 0.76-0.96 |
| ST2b. Specified substitution analyses for processed meat intake ≤ 30 grams/day | | | | | | |
| n= 38 921 | All-cause  (No. of deaths = 4 454) | | Cancer  (No. of deaths = 2 105) | | CVD  (No. of deaths = 531) | |
| Per 20 g/day | HR | 95% CI | HR | 95% CI | HR | 95% CI |
| Lean fish for processed meat | 1.01 | 0.93-1.10 | 1.05 | 0.93-1.19 | 0.92 | 0.72-1.17 |
| Fatty fish for processed meat | 1.06 | 0.97-1.15 | 1.09 | 0.96-1.23 | 0.98 | 0.76-1.26 |

HR, hazard ratio

CI, confidence interval

CVD, cardiovascular disease

Mutually adjusted for red meat, lean and fatty fish, chicken, other fish, shellfish, age (underlying timescale), energy intake (continuous kJ/day excluding energy from alcohol), education (7-9, 10-12, 13-16, ≥ 17 years of schooling), alcohol (non-consumer, 0–5,>5 g/day), smoking (never, current heavy smoker, current moderate smoker, current smoker late starter, former smoker early starter, former smoker late starter), physical activity (low, medium, high), stratified by subcohorts (n=5)

Supplemental Table 3. Hazard ratios (HR) and cause specific mortality according to specified substitution analyses of red meat with lean or fatty fish for women consuming >20 grams/day and ≤ 20 grams/day of red meat starting follow-up two years after baseline

| ST3a. Specified substitution analyses for red meat intake > 20 grams/day | | | | | | |
| --- | --- | --- | --- | --- | --- | --- |
| n= 22 009 | All-cause  (No. of deaths = 2 378) | | Cancer  (No. of deaths = 1 231) | | CVD  (No. of deaths = 260) | |
| Per 20 g/day | HR | 95% CI | HR | 95% CI | HR | 95% CI |
| Lean fish for processed meat | 0.94 | 0.86-1.02 | 1.03 | 0.91-1.16 | 0.92 | 0.71-1.18 |
| Fatty fish for processed meat | 1.00 | 0.91-1.09 | 1.06 | 0.93-1.21 | 1.03 | 0.79-1.35 |
| ST3b. Specified substitution analyses for red meat intake ≤ 20 grams/day | | | | | | |
| n= 55 235 | All-cause  (No. of deaths = 6 017) | | Cancer  (No. of deaths = 2 936) | | CVD  (No. of deaths = 691) | |
| Per 20 g/day | HR | 95% CI | HR | 95% CI | HR | 95% CI |
| Lean fish for processed meat | 0.98 | 0.88-1.10 | 1.00 | 0.85-1.17 | 1.11 | 0.80-1.53 |
| Fatty fish for processed meat | 1.02 | 0.92-1.14 | 1.04 | 0.89-1.21 | 1.17 | 0.85-1.62 |

HR, hazard ratio

CI, confidence interval

CVD, cardiovascular disease

Mutually adjusted for processed meat, lean and fatty fish, chicken, other fish, shellfish, age (underlying timescale), energy intake (continuous kJ/day excluding energy from alcohol), education (7-9, 10-12, 13-16, ≥ 17 years of schooling), alcohol (non-consumer, 0–5,>5 g/day), smoking (never, current heavy smoker, current moderate smoker, current smoker late starter, former smoker early starter, former smoker late starter), physical activity (low, medium, high), stratified by subcohorts (n=5)

Supplemental Table 4. Hazard ratios (HR) and cause specific mortality according to specified substitution analyses of processed meat with lean or fatty fish for women consuming >30 grams/day and ≤ 30 grams/day of processed meat using multiple imputation for missing values on confounding covariates

| ST4a. Specified substitution analyses for processed meat intake > 30 grams/day | | | | | | | | |
| --- | --- | --- | --- | --- | --- | --- | --- | --- |
| n= 49 545 | All-cause mortality (No. of deaths = 5 853) | | | | | | | |
| Per 20 g/day | Model 1a* | | Model 1b† | | Model 2‡ | | Model 3§ | |
|  | HR | 95% CI | HR | 95% CI | HR | 95% CI | HR | 95% CI |
| Lean fish for processed meat | 0.90 | 0.87-0.93 | 0.93 | 0.90-0.96 | 0.93 | 0.90-0.96 | 0.94 | 0.91-0.97 |
| Fatty fish for processed meat | 0.93 | 0.87-0.96 | 0.97 | 0.93-1.00 | 0.97 | 0.93-1.00 | 0.97 | 0.94-1.01 |
|  | Cancer mortality (No. of deaths = 2 910) | | | | | | | |
| Per 20 g/day | Model 1a* | | Model 1b† | | Model 2‡ | | Model 3§ | |
|  | HR | 95% CI | HR | 95% CI | HR | 95% CI | HR | 95% CI |
| Lean fish for processed meat | 0.91 | 0.87-0.96 | 0.94 | 0.90-0.98 | 0.94 | 0.89-0.98 | 0.94 | 0.90-0.98 |
| Fatty fish for processed meat | 0.94 | 0.89-0.99 | 0.97 | 0.92-1.02 | 0.96 | 0.92-1.02 | 0.97 | 0.92-1.02 |
|  | CVD mortality (No. of deaths = 701) | | | | | | | |
| Per 20 g/day | Model 1a* | | Model 1b† | | Model 2‡ | | Model 3§ | |
|  | HR | 95% CI | HR | 95% CI | HR | 95% CI | HR | 95% CI |
| Lean fish for processed meat | 0.80 | 0.74-0.87 | 0.83 | 0.76-0.90 | 0.83 | 0.76-0.90 | 0.85 | 0.78-0.92 |
| Fatty fish for processed meat | 0.84 | 0.77-0.93 | 0.89 | 0.81-0.98 | 0.88 | 0.80-0.97 | 0.90 | 0.82-0.99 |
| ST4b. Specified substitution analyses for processed meat intake ≤ 30 grams/day | | | | | | | | |
| n= 47 912 | All-cause mortality (No. of deaths = 6 455) | | | | | | | |
| Per 20 g/day | Model 1a* | | Model 1b† | | Model 2‡ | | Model 3§ | |
|  | HR | 95% CI | HR | 95% CI | HR | 95% CI | HR | 95% CI |
| Lean fish for processed meat | 0.99 | 0.90-1.04 | 0.98 | 0.92-1.06 | 0.98 | 0.91-1.05 | 0.98 | 0.92-1.05 |
| Fatty fish for processed meat | 1.02 | 0.95-1.09 | 1.03 | 0.96-1.11 | 1.03 | 0.96-1.11 | 1.03 | 0.96-1.11 |
|  | Cancer mortality (No. of deaths = 2 922) | | | | | | | |
| Per 20 g/day | Model 1a* | | Model 1b† | | Model 2‡ | | Model 3§ | |
|  | HR | 95% CI | HR | 95% CI | HR | 95% CI | HR | 95% CI |
| Lean fish for processed meat | 0.94 | 0.85-1.05 | 0.97 | 0.87-1.07 | 0.96 | 0.86-1.07 | 0.96 | 0.86-1.06 |
| Fatty fish for processed meat | 0.99 | 0.89-1.10 | 1.01 | 0.91-1.13 | 1.01 | 0.91-1.13 | 1.01 | 0.91-1.12 |
|  | CVD mortality (No. of deaths = 809) | | | | | | | |
| Per 20 g/day | Model 1a* | | Model 1b† | | Model 2‡ | | Model 3§ | |
|  | HR | 95% CI | HR | 95% CI | HR | 95% CI | HR | 95% CI |
| Lean fish for processed meat | 0.97 | 0.80-1.18 | 0.98 | 0.81-1.20 | 0.97 | 0.80-1.19 | 1.00 | 0.82-1.21 |
| Fatty fish for processed meat | 0.99 | 0.81-1.20 | 1.00 | 0.82-1.22 | 0.99 | 0.81-1.21 | 1.01 | 0.83-1.24 |

HR, hazard ratio

CI, confidence interval

CVD, cardiovascular disease

* Mutually adjusted for lean and fatty fish, chicken, other fish, shellfish, age (underlying timescale), energy intake (continuous kJ/day excluding energy from alcohol), stratified by subcohorts (n=5)

† model 1a + adjusted for education (7-9, 10-12, 13-16, ≥ 17 years of schooling), alcohol (non-consumer, 0–5,>5 g/day), smoking (never, current heavy smoker early starter, current moderate smoker early starter, current smoker late starter, former smoker early starter, former smoker late starter), physical activity (low, medium, high)

‡ model 1b + adjusted for other foods (fruits and vegetables, whole grain products, refined grains, potatoes, dairy products (grams/day continuous))

§ model 2 + adjusted for BMI categories (<20, 20-24.99,25-29.99, >30), type 2 diabetes

# Supplemental Figures

Supplemental Figure 1. Directed acyclic graph (DAG) illustrating the hypothesized causal relationships between covariates in the association between the substitution of red and processed meat with lean or fatty fish and mortality

| 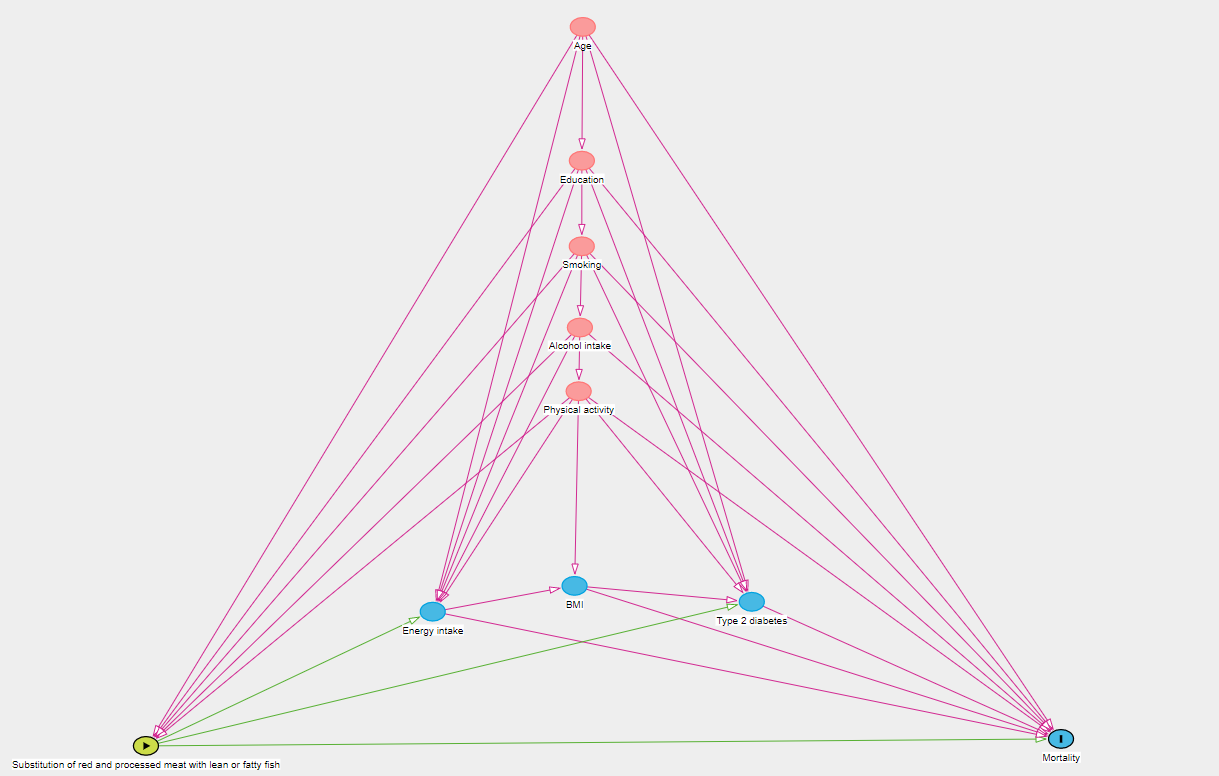 |  |
| --- | --- |


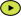
 exposure
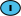
 outcome
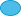
 ancestor of outcome
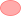
 ancestor of exposure and outcome
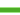
 causal path
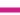
 biasing path

BMI= body mass index

The DAG illustrates the hypothesized causal relationships between covariates in the association between the substitution of red and processed meat with lean or fatty fish and mortality. The direction of the arrows illustrates the assumed direction of the causal relationship between the covariates and the exposure and outcome. The arrows direction is based on the following assumptions:

Increasing age, smoking, and high alcohol intake are associated with higher mortality, while higher levels of education and physical activity are linked to lower mortality.

Age-related changes, along with the influence of education, smoking, alcohol consumption and physical activity, impact food intake, food choices, and other lifestyle factors. Smoking is associated with higher red and processed meat intake, alcohol consumption, and a person's level of physical activity. As these factors affects both the exposure and outcome as illustrated in the DAG, they are identified as confounders in the relationship between substitution of red and processed meat with lean or fatty fish and mortality.

Food and energy intake play a role in BMI, BMI is also a risk factor of type 2 diabetes. Furthermore, processed meat consumption has been linked to a higher risk of type 2 diabetes, while the intake of lean fish has been found to reduce the risk of type 2 diabetes. Both BMI and type 2 diabetes are associated with mortality and are thus considered mediators in the hypothesized causal pathway between substituting red and processed meat with lean or fatty fish meat and mortality. However, it's important to note that BMI is also related to energy needs and energy expenditure, which can lead to increased food and energy intake in individuals with higher BMI. Additionally, being diagnosed with type 2 diabetes may induce dietary changes. Therefore, both BMI and type 2 diabetes can act as mediators as illustrated in the DAG (Substitution of red and processed meat with lean or fatty fish → BMI → type 2 diabetes → mortality) or confounders (Substitution of red and processed meat with lean or fatty fish ← BMI / type 2 diabetes → mortality) in the relationship between substitution of red and processed meat with lean or fatty fish and mortality.

##

Supplemental Figure 2. Intake of red and processed meat and cause specific mortality by restricted cubic spline regression

HR

| All-cause mortality | Cancer mortality | CVD mortality |
| --- | --- | --- |
|   HR  gram intake |   HR  gram intake |   gram intake |

HR, hazard ratio

CVD, cardiovascular disease

Black line hazard ratio, gray area 95% confidence interval, p-value for non-linear trend.

Red and processed meat intake modelled using restricted cubic splines with three knots at percentiles 10%, 50% and 90% (18, 45, 84 grams/day), 50 grams ref. value.

Mutually adjusted for lean fish, fatty fish, chicken, other fish, shell fish, age (underlying timescale), energy intake (continuous kJ/day excluding energy from alcohol), education (7-9, 10-12, 13-16, ≥ 17 years of schooling), alcohol (non-consumer, 0–5,>5 g/day), smoking (never, current heavy smoker, current moderate smoker, current smoker late starter, former smoker early starter, former smoker late starter), physical activity (low, medium, high), stratified by subcohorts (n=5) (model 1b)
